# Fenugreek derived diosgenin as an emerging source for diabetic therapy

**DOI:** 10.3389/fnut.2024.1280100

**Published:** 2024-02-02

**Authors:** Yamini Tak, Manpreet Kaur, Abhishek Chitranashi, Mahesh Kumar Samota, Preeti Verma, Manoj Bali, Chiranjeev Kumawat

**Affiliations:** ^1^Agricultural Research Station, Agriculture University, Kota, India; ^2^Department of Biochemistry, Punjab Agricultural University, Ludhiana, India; ^3^Division of Biochemistry, ICAR-Indian Agricultural Research Institute, New Delhi, India; ^4^ICAR-Central Institute of Post-Harvest Engineering & Technology, Ludhiana, India; ^5^School of Sciences, Rayat Bahra University, Mohali, India; ^6^Sri Karan Narendra Agriculture University, Jaipur, India

**Keywords:** bioactive, diabetes, diosgenin, extraction, fenugreek

## Abstract

Diabetes is a chronic metabolic disease that endangers the entire body’s tissues and organs. Diabetes impairs glucose and insulin regulation in the human body by causing pancreatic cell damage. Diabetes modifies pathways such as serine/threonine protein kinase (Akt) and Protein kinase C (PKC)/- glucose transporter 4 (GLUT4), peroxisome proliferator-activated receptor (PPAR) glucose absorption, and inhibits α-amylase and α-glucosidase, Sodium/glucose cotransporter 1 (SGLT-1), and Na^+^-K^+^-ATPase activity. Diabetes may also be caused by a decrease in the expression of sterol regulatory element binding protein 1 (SREBP-1) and its target genes, fatty acid synthase (FAS), stearoyl-CoA desaturase-1 (SCD-1), and acetyl-CoA carboxylase α (ACC), as well as a decrease in the levels of C/EBP homologous protein (CHOP), Caspase12, and Caspase3 proteins. Diabetes has long been linked to diseases of the cardiovascular, nervous, skeletal, reproductive, hepatic, ocular, and renal systems. Diosgenin, a steroidal compound derived from fenugreek, aids in the prevention of diabetes by altering cellular pathways in favor of healthy bodily functions. Diosgenin is a new nutraceutical on the market that claims to cure diabetes in particular. This article focuses on diosgenin extraction and purification, fenugreek bioactive compounds, pharmacological properties of diosgenin, mode of action of diosgenin to cure diabetes, and dosages.

## Graphical Abstract

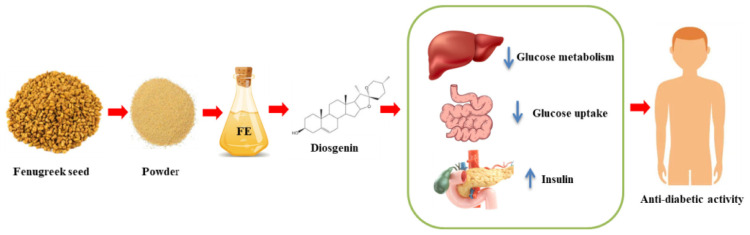

## Introduction

Due to promising results and rare side effects, medicinal herbs have recently received a lot of attention across the world for their use in the treatment of various ailments. Phytochemicals found in herbal medicines, such as phenolic acids, saponins, flavonoids, tannins, alkaloids, and terpenoids, aid in the treatment of human disease. Fenugreek (*Trigonella foenum-graecum* L.) is an ancient or traditional remedial plant is one of the evidence-based herbal treatments (1). Fenugreek, also known as Methi, Chandrika, Alholva, Bird’s Foot, Bockshornsame, and Greek Clover, is a member of the Fabaceae family that originated in India and Northern Africa and is now commercially grown in

Mediterranean Europe, China, Southeast Asia, Australia, the United States, Argentina, and Canada. India is one of the world’s greatest fenugreek growers, yet it does not have a significant proportion of the worldwide fenugreek trade due to high internal consumption. Fenugreek seeds and leaves have been used for millennia in Indian, Tibetan, and Chinese medicine to treat a variety of ailments, including diabetes, obesity, polycystic ovarian syndrome, atherosclerosis, cancer, inflammation, and blood cholesterol (2, 3). The excellent nutritional profile and bioactive components in fenugreek give it medicinal and pharmacological properties including antibacterial, anticholesterolemic, carminative, restorative, uterine tonic, anti-carcinogenic, anti-inflammatory, antiviral, antioxidant, and hypotensive effects (4). Essential oil, coumarins, alkaloids (trigonelline, isoorientin), polyphenols (rhaponticin, isovitexin), and steroidal saponins (diosgenin) are all found in fenugreek seed extract (5, 6). The most active antidiabetic bioactive found in fenugreek is diosgenin, which possesses antioxidative properties (7).

According to recent data from the United States, prediabetes affects 34.6% of the adult population, whereas impaired fasting glucose (IFG) affects 19.4%, impaired glucose tolerance (IGT) affects 5.4%, and IFG plus IGT affect 9.8% (8). In India, according to ICMR 11.4% population living with diabetes whereas, 15.3% of the population is prediabetic. IGT is believed to have 316 million users worldwide, with that number expected to climb to 471 million by 2035 (9). Persons with diabetes have a 7-year reduction in life expectancy than the general non-diabetic population, an effect that is intimately linked to the most serious diabetic outcomes, which include heart disease, limb amputations, End-Stage Renal Disease, and blindness (10). Currently used chemotherapeutic mediators have shown to be helpful in the treatment of diabetes, but they come with a slew of unpleasant side effects, including appetite loss, stomach discomfort, muscle cramps, and weakness. Diosgenin has antioxidative properties and aids in the treatment of diabetes through a variety of mechanisms, including β-cell renewal and insulin secretion stimulation by increasing CCAAT/enhancer-binding protein (C/EBP δ) and peroxisome proliferator-activated receptor- γ (PPAR- γ) mRNA transcription levels (11). Chemotherapeutic mediators used today have shown to be quite helpful in the treatment of diabetes; however, they have a number of unfavorable side effects such as loss of appetite, abdominal discomfort, muscle cramping, and weakness. This review article seeks to make the most of the information available to highlight the topic’s scientific standing and the requirement for new research in order to improve current knowledge since more study is currently needed in this area.

## Diabetes types and issues

Diabetes is a chronic disease in which the pancreas is unable to produce insulin or the human body is unable to use the insulin produced effectively, resulting in an imbalance in glucose metabolism or an increase in blood sugar levels. According to American Diabetes Association 2009 (12), there are mainly diabetes types are: Type 1 diabetes, type 2 diabetes, type 3 diabetes, and gestational diabetes. Diabetes also causes other serious health problems and is a significant financial burden for a vast number of people around the world. Poor nutritional diets and unhealthy or contemporary lifestyles contribute to weight gain and obesity, which can be a contributing factor to diabetes around the world. According to the World Health Organization (WHO), diabetes affects around 422 million people globally, the majority of whom live in low- and middle-income countries. Diabetes or higher-than-optimal blood glucose caused 1.5 million individuals to die directly from cardiovascular disease, chronic renal disease, or tuberculosis in 2019. According to the American Diabetes Association (ADA) (13), almost 1.9 million Americans, including roughly 2.44 lakh children and adolescents, have type 1 diabetes. In 2017, the entire cost of diabetes was $327 billion, with $237 billion in direct medical costs and $90 billion in indirect costs due to lost productivity. Type 1 diabetes is a polygenic inherited disease caused by a complicated interplay between the pancreatic β-cell and the innate and adaptive immune systems. Insulin is not generated as a result of destructed- β cell and the human body is unable to store excess glucose, resulting in an increase in blood sugar levels. Hypoglycemia is a complication of Type 1 diabetes that causes confusion, convulsions, or coma in the patient. Individuals with Type 1 diabetes can benefit from continuous subcutaneous insulin infusions, oral medication, and a nutritious diet combined with a healthy lifestyle, thyroid dysfunction treatment, and glucose self-monitoring (14, 15).

Globally, Diabetes mellitus affects approximately one in every eleven adults, with Type 2 diabetes accounting for 90% of cases. Asia is a chief region of the world’s rapidly spreading Type 2 diabetes mellitus epidemic, with China and India serving as the top two epicenters (16). Type 2 diabetes is caused by high blood sugar levels. Blood glucose is your primary source of energy, and it is derived primarily from the foods we consume. In Type 2 diabetes, the body either does not produce enough insulin or does not use insulin effectively. Type 2 diabetes affects more than 90% of diabetic patients, causing microvascular and macrovascular complications that cause profound psychological and physical distress in patients, as well as a significant burden on health-care systems. Overweight and obesity, a lack of physical activity, insulin resistance, and genetic factors all contribute to Type 2 diabetes (17). Insulin resistance is linked to a slew of metabolic issues, including glucose intolerance, hypertension, a distinct dyslipidemia, a procoagulant state, and an upsurge in macrovascular disease (18). Cardiovascular disease is the leading cause of illness and death in Type 2 diabetes and necessitates close monitoring of glucose and lipid levels, as well as blood pressure, to reduce the risk of complications and disease progression (19). Increased thirst, frequent urination, increased hunger, weight loss, blurred vision, slow-healing sores, and frequent infections are common symptoms of Type 2 diabetes.

Gestational diabetes is a type of glucose intolerance that develops during pregnancy as a result of insufficient insulin supply to meet tissue demands for standard blood glucose regulation (20). Gestational diabetes appears to be caused by the same wide range of physiological and genetic abnormalities that characterize diabetes in general. Maternal overweight and obesity, later childbearing age, previous history of Gestational diabetes, family history of type 2 diabetes mellitus, and ethnicity are all major risk factors for Gestational diabetes (21). In the United States, African American, Hispanic American, Native American, Pacific Islander, and South or East Asian women have a higher prevalence than Caucasian women (22). According to a recent International Diabetes Federation report, 16% of live births worldwide in 2013 were complicated by hyperglycemia during pregnancy (23). According to the most recent meta-analysis by Saeedi et al. (24), the global prevalence of GDM is 14.7% based on the International Association of Diabetes and Pregnancy Study Groups (IADPSG) criteria, which is the most widely used screening method worldwide. Pregnancy diabetes can result in problems such as the baby growing larger than usual, polyhydramnios, premature birth, pre-eclampsia, and infant jaundice.

## Fenugreek’s cultural and historical importance

Fenugreek is culturally significant Ayurvedic medicine around the world, including India, Egypt, and the Middle East. Fenugreek is a spice that is widely used in India and the Mediterranean region and is recognized to have a variety of medicinal properties. Fenugreek seeds are widely used in Indian cuisine and are an essential ingredient in curry powders. They are also used to enhance the flavour of pickles and chutneys (25). Fenugreek leaves are commonly used in salads and traditional Egyptian dishes such as Ful medames. Fenugreek seeds are used as a spice in many Middle Eastern dishes, including meat and vegetable dishes. In recent years, selected genotypes of this species have developed a niche crop that produces high yields of bloat-free forage, which can boost both beef and milk production in semiarid regions of western Canada. Fenugreek has cultural significance in Jewish tradition, where it is used during the Sukkot festival. The herb is one of four plant species that are gathered and waved during prayers. Fenugreek is also important in Islamic culture, where it is used in cooking and medicine.

Fenugreek has a long history dating back to antiquity. The herb was used for its medicinal properties in ancient Egypt and was thought to have been used by Cleopatra to enhance her beauty. Fenugreek was used to treat a variety of ailments in ancient Greece, including digestive issues, respiratory problems, and skin inflammation. Fenugreek was also used extensively in ancient Ayurvedic medicine in India. The herb was thought to have healing properties that could be used to treat a variety of health issues, including diabetes, inflammation, and digestive disorders (26). Fenugreek was also used in traditional Chinese medicine to treat various conditions, including asthma and digestive problems. In addition to its medicinal uses, Fenugreek was also used for religious and cultural purposes in ancient times. In ancient Egypt, Fenugreek was used in religious ceremonies and was believed to have healing properties. In ancient Rome, Fenugreek was used as a flavouring agent in food and was also used in perfumes and cosmetics. Fenugreek is still used for its medicinal and culinary properties today. It is thought to have a variety of health benefits, including lowering blood sugar levels, reducing inflammation (27), and increasing milk production in breastfeeding mothers National Institute of Child Health and Human Development (28). Fenugreek is also a popular ingredient in bodybuilding supplements because it is thought to boost testosterone levels (29).

## Bioactive compounds of fenugreek seeds and their pharmacological property

Fenugreek seeds are high in bioactive compounds such as saponins, alkaloids, flavonoids, and phenolic compounds. At this point, we will look at fenugreek’s bioactive compounds and their potential health benefits (Table 1). Secondary metabolites found in fenugreek seed include saponin (4.8%), flavonoids (100°mg/gm), alkaloids (35%), and diosgenin (0.2−0.9%) (30). Among these, alkaloids are primarily responsible for the distinctive taste and aroma. Saponins, which are glycosides with strong foam-forming properties, are abundant in fenugreek seeds. Saponins are thought to have a variety of health benefits, including cholesterol-lowering and anticancer properties. Saponins have been shown in studies to bind to bile acids in the intestine and prevent their reabsorption, resulting in lower cholesterol levels. Saponins have also been shown *in vitro* to inhibit cancer cell growth and induce apoptosis (programmed cell death) (31).

**TABLE 1:** Bioactive compounds of fenugreek (leaves and seed).

Class	Bioactive compound	Bioactivity	References
Sapogenin	3,5-Spirostadiene derivative, Smilagenin, Sarsasapogenin, Diosgenin (0.1–0.9%), Tigogenin, Yamogenin, Neotigogenin, Yuccagenin, Gitogenin, Neogitogenin	Antinflammatory, antidiabetic, neuroprotectiver, anticancerous, hypolipidemic effect	(37–39)
Saponin	Graecunins, Fenugrin B, Fenugreekine, Trigofoenosides A–G	−	(39)
Flavonoids	Naringenin, Quercetin, Tricin, Kaempferol, Luteolin, Quercitrin, Afroside, Isovitexin, Vitexin, Vicenin-1, Vicenin-2	antidiabetic, antiatherogenic, antidepressant, immunomodulatory, antitumor, anti-inflammatory, anticancer	(40–42)
Phenolics and their derivatives	p-Coumaric acid, Caffeic acid, Chlorogenic acid, Hymecromone, Trigoforin, Trigocoumarin, Scopoletin, gamma-Schizandrin, Zingerone, Vanillin, Gingerol, Eugenol	antidiabetic, antihyperlipidemic, antiobesity, anticancer, anti-inflammatory, antioxidant, antifungal, antibacterial	(2, 42)
Alkaloid	Trigonelline, N-methylnicotinic acid, Trimethylamine, Neurin, Choline, Gentianine, Carpaine, and Betain	hypoglycaemic, neuroprotective, anti-cancer, estrogenic, and antibacterial activities	(2, 43)
Lipids	Oleic, Linoleic, Linolenic acids, Phosphatidylcholine, Phosphatidyl ethanolamine	anti-cancer, anti-inflammatory	(2, 42)
Vitamin and minerals	Vitamins: A, B_1_, B_2_, C, niacin, nicotinic acid, β-carotene, Folic acid Minerals: Fe, Ca, P, S, Mg, Co, Cu, Mn, Zn, Br	−	(25)
Dietary fiber	Galactomannan	Reduce obesity and improve gut health	(44)

Trigonelline, gentianine, and carpaine are among the alkaloids found in fenugreek seeds. Trigonelline is an extremely powerful antioxidant that has been shown to protect against oxidative stress and DNA damage (32). Gentianine has anti-inflammatory and analgesic properties, whereas carpaine has hypotensive (blood pressure-lowering) properties. Flavonoids, which are polyphenolic compounds with antioxidant properties, are abundant in fenugreek seeds (33). The flavonoids vitexin and isovitexin are the most abundant in fenugreek seeds. Vitexin has been shown to have anti-diabetic properties by increasing insulin sensitivity and decreasing blood glucose levels. Isovitexin has been shown to have neuroprotective properties and may aid in the prevention of cognitive decline (34). Fenugreek seeds contain phenolic compounds such as coumarins and lignans. Coumarins have anticoagulant properties and may aid in the prevention of blood clots. Lignans have anticancer properties and may help reduce the risk of breast cancer (35).

Other bioactive compounds found in fenugreek seeds include galactomannans, mucilages, and phytosterols. Galactomannans are complex carbohydrates that have been shown to lower cholesterol levels. Mucilages are water-soluble fibres that have been shown to have prebiotic properties and may aid in the improvement of digestive health (36). Phytosterols are plant-based compounds that have been shown to lower cholesterol levels. It contains a lot of dietary fibre, protein, amino acids, iron, silica, and vitamin B1. Furthermore, fenugreek seed contains an unusual amino acid called hydroxy isoleucine, which increases insulin secretion and helps to prevent diabetes (11).

## Diosgenin extraction, purification, and characterization

The most active bioactive ingredient in fenugreek seed extract is diosgenin [0.113−0.135% (w/w)], which has anti-diabetic properties. The melavonate route, which comprises of a hydrophilic sugar moiety attached to a hydrophobic steroid aglycone, is used to produce diosgenin. When fenugreek seed was extracted using 70% (v/v) 2-propanol in water and sulphuric acid, the extract included a mixture of diosgenin with steroidal saponins, and other slight sapogenins (11). Diosgenin can be extracted from *Dioscorea nipponica* Makino using a magnetic sulfonated solid composite and hydrolyzing it with 2.5°M hydrochloric acid at 110°C for 5°h (45). Green extraction technologies such as ultrasound-assisted extraction (UAE) and microwave-assisted extraction (MAE) can extract the most diosgenin from fenugreek seeds, which can then be used to treat diabetes patients. For diosgenin extraction, the MAE method used a sample-to-solvent ratio is 1:5 (w/v) with solvents (acetone, ethanol, hexane, and petroleum ether) and extraction time (1.5, 3.0, 4.5, and 6.0°min) at 180 W power to yield 7.83% diosgenin content. In the UAE approach, 21.48% diosgenin was obtained when the sample-to-solvent ratio was 1:5 (w/v) with different solvents, treatment times were varied (30, 40, 50, and 60 min), and the ultrasonic bath temperature was prolonged at 30°C throughout the procedure (46). Likewise, deoiled fenugreek seed powder (20 g) was extracted with 100 ml ethanol (20−100%,v/v), extraction time 40, 50, and 60 min, and the temperature of the ultrasonic bath throughout the extraction process was 35°C to detect diosgenin (0.041−1.294 geq/100 g) (47). UAE method showed highest diosgenin from fenugreek seeds with 80% ethanol for 5 min, with α-amylase inhibition (IC_50_ crude = 371.7°μg/ml, IC_50_ defat = 370.5°μg/ml) and pancreatic lipase (IC_50_ crude = 550.0°μg/ml and IC_50_ defat = 497.6°μg/ml). For purification dehydrated fenugreek seed extract was dissolved in distilled water (50 ml), flushed twice with diethyl ether (50 mL), and extract liquid layer was extracted with water-saturated n-butanol (50 mL). Diosgenin profiles were constructed for seed samples of various fenugreek varieties, and the results revealed 200 and 480 mg/100 g FW diosgenin concentrations (48).

## Chemical structure, health benefits, and worldwide status of diosgenin

**FIGURE 1:**
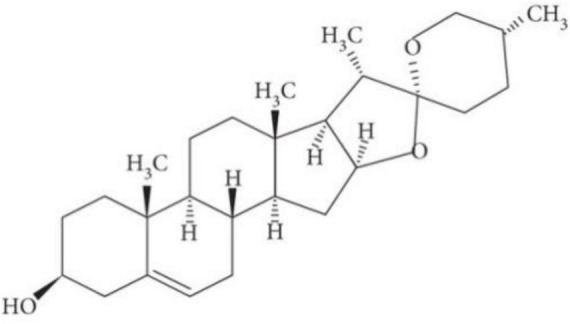
Chemical structure of diosgenin (53).

Diosgenin is a major bioactive constituent of many edible pulses and roots, particularly in the seeds of fenugreek and the root tubers of wild yams. Diosgenin is found in 137 different Dioscorea species. A total of 41 of them contain more than 1% diosgenin. It is derived from the roots of the Dioscorea wild yam that is commonly used as a precursor in the production of synthetic steroid chemicals such as progesterone and cortisol (49). According to the National Center for Biotechnology Information 2023 (50), Diosgenin (25*R*-spirost-en-3β-ol) is a C27 triterpenoid spiroketal steroid sapogenin with a molecular weight of 414.62 and its formula is C_27_H_42_O_3_ (Figure 1). It is described as a spirostan with a hydroxyl group at the β position in terms of molecular structure. It also contains a double bond at 5,6 position and has an R configuration at position 25. It has a hydroxyl group in the third position; hydroxyl groups are typically found in combination with sugars, making the compounds water-soluble and highly saponaceous (51). In aqueous medium, it has a solubility of about 0.7°ng/ml. It is a white crystalline powder that dissolves in organic solvents such as ethanol, DMSA, and dimethylformamide. Cholesterol is a precursor in the biosynthesis of diosgenin, which is catalysed by two P450 enzymes: C-16,22-dihydroxylase and C-26 hydroxylase (52).

In addition to being an important starting material for the preparation of several steroidal drugs in the pharmaceutical industry, diosgenin has shown high potential and interest in the treatment of various disorders such as cancer, diabetes, arthritis, asthma, and cardiovascular disease (54). Diosgenin supplementation is thought to be an excellent way to promote women’s health because it slows the decline of estrogen and progesterone levels and aids in the prevention of hormonal imbalances. In essence, it may reduce the risk of osteoporosis, mood swings, irritability, and other symptoms associated with fluctuating hormone levels (55). Furthermore, research suggests that Diosgenin supplementation may help gastroprotection against stomach mucosa damage by inhibiting certain enzyme activity (56).

Because of the COVID-19 pandemic, the global Diosgenin market is estimated to be worth USD 99.5 million in 2022 and is expected to grow to USD 142.3 million by 2030, with a CAGR of 6.1% from 2022 to 2030. According to a Press Release of Diosgenin Market Size by 2030, Diosgenin accounts for 60% of the world’s steroidal products out of all steroid drug precursors. With a market share of roughly 60%, China is the world’s largest diosgenin consumer market. Sabinsa, Himachal Pharmaceuticals, Namiex Chemicals, Zhenhua Biology, and Shaanxi Jiahe Biotechnology are the global top five diosgenin manufacturers, with a combined market share of more than 85%, with Zhenhua Bio being the largest manufacturer, with a market share of more than 55%.

## Modes of action of fenugreek derived diosgenin in diabetes

**FIGURE 2:**
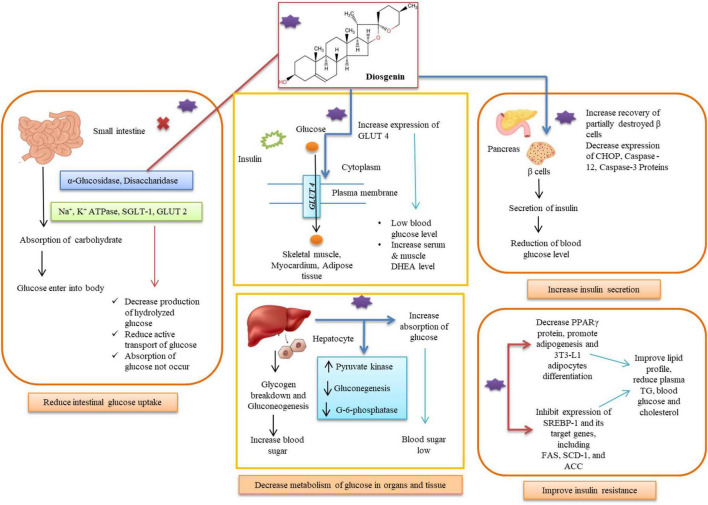
Diosgenin for diabetes treatment by multiple pathways in different organs.

Numerous researches have looked into the effects of fenugreek extracts and diosgenin in the treatment of diabetes and their mechanisms of action with possible advantages for diabetics (Table 2). These consist of clinical trials, *in vitro* and *in vivo* studies (Figure 2). Further explanations of the processes, outcomes, and diosgenin’s mode of action are provided in this section.

**TABLE 2:** *In vitro*, *in vivo* studies and mechanism of action for treating diabetes with fenugreek and diosgenin.

Plant/Part and Location	Bioactive compound/Extract	Study	Clinical trial	Mode of action/Inference	Reference
*Trillium govanianum*, India	Sitagliptin, Diosgenin, Borassoside, Protodioscin	*In vitro*	−	Borassoside E (IC50 7.15 ± 1.78°μM), protodioscin (IC50 6.72 ± 0.04°μM), and diosgenin (IC50 12.75 ± 2.70°μM) inhibiting the activity of α-amylase, α-glucosidase, and dipeptidyl peptidase IV, respectively.	(100)
*Gnidia glauca* (Leaves, stems and flowers), *Dioscorea bulbifera* (Bulbs) Maharashtra, India	Petroleum ether, ethyl acetate and methanol extracts of plant	*In vitro*	−	Petroleum ether extract of *G. glauca* flower inhibit α-amylase by 78.56% and ethyl acetate extract of *D. bulbifera* bulbs inhibit α-amylase by 73.39%. Can use to treat type II diabetes mellitus.	(101)
Fenugreek (*Trigonella foenum-graecum* L. seed), London	Crude ethanolic extract B, further sub-fractions of B (saponin-free C, saponin D and sapogenin E) and a gum fiber fraction F	*In vitro*	−	Inhibited glucose-uptake at 0.33 and/or 3.3°mg/mL (*p* < 0.001).	(63)
Fenugreek (*Trigonella foenumgraecum*) seeds, India	80% ethanol by UAE method for 5 min produced 13.78 g diosgenin equivalent/100 g	*In vitro*	−	Inhibit α-amylase (IC50 crude = 371.7°μg/ml, IC50 defat = 370.5°μg/ml). Diosgenin assist in delaying glucose diffusion.	(48)
Fenugreek seed and Balanites fruit, Egypt	Methanolic extract	α-amylase (*in vitro*), starch absorption (*in vivo*)	STZ-diabetic rats (50 mg/kg) normal (*n* = 10) and diabetic groups (*n* = 45)	Fenugreek extract administrated STZ-diabetic mice, and decreased liver glucose-6-phosphatase activity, restored liver glycogen content, cut blood glucose levels by 58% and inhibit intestinal α-amylase activity.	(102)
*Trigonella foenum-graecum*	Saponin fraction contains diosgenin	*In vivo*	Four-week-old male KK-Ay/Ta Jcl mice administrated high-fat diet supplemented with 0.5 or 2.0% fenugreek	In HepG2 cells, diosgenin (5 and 10°μmol/L) reduced TG accumulation and the expression of genes related to lipogenesis and helpful for the controlling of diabetes-related hepatic dyslipidemias.	(103)
Fenugreek seed, Hungary	Diosgenin	*In vivo*	Diosgenin in three different doses (1°mg/bw kg, 10°mg/bw kg, and 50 mg/bw kg) and fenugreek seed (0.2 g/bw kg) were administered orally for 6 weeks to Male Wistar rats.	Rats given 1 mg/kg diosgenin with fenugreek seed showed better peripheral insulin sensitivity as evidenced by higher insulin sensitivity index and high metabolic clearance rates of insulin. Fenugreek seed regulates hormones in synchronised action with IGF-1, which is crucial for maintaining appropriate blood sugar levels.	(95)
Fenugreek seed, Puducherry, India	Methanolic extract contain trigonelline and diosgenin	*In vivo*	Male Sprague–Dawley rats (8−10 weeks with 150−200 g BW) administering STZ (dose of 35 mg/kg BW). Group 1 = control (NPD), Group 2 = T2DM model rats (HFD followed by STZ administration), Group 3, 4, 5 T2DM rats were given 300 mg FSE/kg BW, 40 mg of trigonelline/kg BW, 60 mg of diosgenin/kg BW, respectively.	Rats treated with FSE (217.11 ± 2.36 mg/dL), trigonelline (275.13 ± 24.88 mg/dL), and diosgenin (218.32 ± 37.9 mg/dL) showed significant reduction in glucose levels as compared to T2DM group (506.94 ± 7.06 mg/dL). T2DM rats showed two- to threefold increase in ER chaperones Bip, protein disulfide isomerase (PDI), as well as ER stress-related proapoptotic markers CHOP, Caspase12, and Caspase3 in the liver, and increased lipid peroxidation (LPO) and decreased antioxidant levels.	(104)
India	Diosgenin	*In vivo*	Male albino Wistar rats (BW = 150−180g) fed with HFD and administered with STZ (35 mg kg^–1^), Group 1: Control, Group 2: Diabetic control, Group 3: Diabetic rats administered with diosgenin (60 mg kg^–1^b.w^–1^), Group 4: Diabetic rats administered with glyclazide (5 mg kg^–1^ b.w^–1^).	Administration of diosgenin to HFD-STZ diabetic rats depicted a decrease in body weight gain, blood glucose (118.9 ± 9.92 mg/dL), insulin (14.65 ± 2.4°μU ml^–1^), insulin resistance (2.01 ± 0.32) and modulated lipid profile in plasma and tissues as compared to HFD-STZ Diabetic control rats.	(105)
Fenugreek, India	Diosgenin	*In vivo*	Male Wistar rats (BW = 150−180 g) administrated 55 mg/kg streptozotocin with three different doses (15, 30, and 60 mg/kg BW) of diosgenin	Rats given 30 mg/kg BW diosgenin had lower serum Glucose 6-phosphatase levels (106.1 ± 2.9) compared to diabetic rats (155.0 ± 6.3) and higher hexokinase levels (15.8 ± 2.03) compared to diabetic rats (8.09 ± 2.1), which improved glycogen metabolism and lowered blood glucose levels.	(84)
China	Diosgenin	*In vivo*	Female mice were administered orally with DSG (10 mg/kg b.w. and 20 mg/kg b.w) using an intra-gastric tube	Diosgenin decreases gestational diabetes in db/ + pregnant mice, by improvements in glucose and insulin resistance, a decline in fasting blood glucose and insulin levels, and an increase in hepatic glycogen content.	(106)
Fenugreek seed, India	−	*In vivo*	Sixty patients having Type 2 diabetes divided into two groups. Group 1 = 30 patients received 10°gm of fenugreek seeds soaked in hot water every day, Group 2 = 30 patients didn’t received fenugreek seeds.	Reduction in fasting blood glucose levels in the 5th month in the study group (*P* = 0.0421) while significant reduction in HbA_1_C in the 6th month (*P* = 0.0201)	(107)

## Diminish glucose absorption in intestine

The pancreatic β-cells’ ability to secrete insulin is reduced by hyperglycemia in the toxicity cycle, which is then followed by a rise in insulin resistance, which worsens hyperglycemia and renders β -cells completely ineffective. The long-lasting and serious health repercussions of hyperglycemia take time to manifest (57). The small intestine has the ability to absorb carbohydrates, which allows glucose to enter the bloodstream and easily raises postprandial hyperglycemia in diabetics. Polysaccharides are primarily broken down into oligosaccharides in the luminal bulk fluid by secreted enzymes such as α-Glucosidase, disaccharidases, Na^+^-K^+^-ATPase, and additional hydrolysis is carried out by an array of carbohydrases in the brush border of the mature enterocytes (58). The Na^+^-glucose cotransporter SGLT1 actively transports glucose and galactose into the enterocyte via the transmembrane electrochemical Na^+^ gradient, whereas the glucose transporter GLUT2 actively transports glucose and galactose out across the basolateral membrane (59). Diosgenin showed a considerable amount of α -amylase and α -glucosidase inhibitory impact, supporting its involvement in lowering high blood glucose levels. Only one catalytic residue from α-amylase is involved in hydrogen bonding contact with diosgenin, while diosgenin interacts with two catalytic residues from α-glucosidase (Asp352 and Glu411) to generate the lowest energy inhibitor complex (60). Other hydrophobic interactions, in addition to hydrogen bonding interactions, contributed to the maximum binding affinity of diosgenin toward α-glucosidase. 33 steroidal saponins and sapogenins were extracted from fenugreek, and their *in vitro* α -glucosidase inhibitory action was assessed (61). There were five 25R and 25S isomer combinations of spirostanol saponins or sapogenins among them, and these were compounds 10 (25R/S)-5α-spirostane-2α,3β-diol 3-O-α-L-rhamnopyranosyl- (1 → 2)-β-D-glucopyranoside, 12 diosgenin/yamogenin, 17 (25R/S)-5-en-spirostane-3β-ol 3-O-α-L-rhamnopyranosyl-(1 → 2)-β-D-glucopyranoside, 22 (25R/S)-5-enspirostane-2α,3β-diol 3-O-α-L-rhamnopyranosyl-(1 → 2)-β-D-glucopyranoside, and 29 sarsasapogeninn/smilagenin. Saponins 18 (25R)-5-en-spirostane-3β-ol 3-O-β-Dglucopyranosyl-(1 → 4)-β-D-glucopyranoside, 23 (25R)-5-en-spirostane-2α,3β-diol 3-O-α-L-rhamnopyranosyl-(1 → 2)-[α-L-rhamnopyranosyl-(1 → 4)]-β-D-glucopyranoside, 26 soyasapogenol B, 27 3-O-β-D-glucuronopyranosyl soyasapogenol B methyl ester, and 14 isonarthogenin significantly inhibited α-glucosidase at IC_50_ values of 15.16, 8.98, 7.26, 5.49, and 14.01°M, respectively, as compared to the positive control. Lactase and maltase activity in the gut of diabetic rats was significantly reduced by sapogenin extract or diosgenin supplementation. Diosgenin consumption revealed hypoglycemic qualities that are advantageous in diabetes by lowering intestinal disaccharidases activity. Diabetic rats’ Na^+^-K^+^-ATPase activity was shown to be drastically decreased when diosgenin was added to their diet. Supplementation of the diet with the 1% commercial diosgenin significantly reduced Na^+^-K^+^-ATPase activity in all three regions proximal (12.8 ± 0.2°nmol Pi/min/mg protein), mid (9.5 ± 0.1°nmol Pi/min/mg protein), distal (7.3 ± 0.1°nmol Pi/min/mg protein) of the intestine of the diabetic control rats when compared to the normal control group compared to the diabetic control group (62). Comparing the diabetes control to the diosgenin-supplemented group, the proximal area of the body showed a significantly higher level of Ca^2+^ ATPase activity. Sapogenin found in fenugreek extract reduced glucose absorption at concentrations of 0.33 and/or 3.3 mg/mL (*p* < 0.001). A total of 1 kg Fenugreek seed was ground and continuously extracted with light petroleum for 16 h and dried by rotary evaporation to yield an oil fraction (A; 45°g). Fenugreek seed powder (895°g), which had been defatted, was dried at room temperature and subjected to a 24-hour continuous extraction with 100% ethanol. The crude ethanolic extract (B; 65°g) was obtained after drying as before. A portion of B was redissolved in water and further extracted by n-butanol (C; 16.9°g) by evaporating the n-butanol layer, leaving the water layer containing saponin-free extract (D; 13.7°g on freeze drying). Diosgenin and trigonelline both prevented the absorption of glucose, with IC_50_ values of nearly 8 and 19 mM, respectively. Diosgenin (1.65 mg/ml) was more efficient than trigonelline (9.4% non-significant inhibition at 1.65 mg/ml) in inhibiting glucagon-induced HGPa activity (63). Diosgenin, which shares structural similarities with dehydroepiandrosterone (DHEA), reduces hyperglycemia in streptozotocin (STZ)-induced type 1 diabetes mellitus mice through increasing muscle GLUT4 signalling. After receiving diosgenin injection for 120 min, serum DHEA levels dramatically increased; concurrently, blood glucose levels significantly dropped (64).

## Inhibit glucose uptake

It’s crucial to comprehend the mechanism through which organs and tissues absorb glucose. Inhibiting hyperglycemia and its associated consequences can be prevented in the ideal way thanks to this information. Since most live cells in the body require the transportation of glucose, it is a primary source of energy for mammalian cells. Every cell’s internal structure is protected by a phospholipid bilayer (65). Glucose molecules must cross lipid bilayers in the cell membrane as part of the transportation mechanism. Passive diffusion is used to allow hydrophobic species to travel across the hydrophobic lipid bilayers, which are permeable to them. Contrarily, glucose is hydrophilic and travels through the bilayer via enhanced diffusion, which is made possible by the career protein. Although the precise structure of the career protein for glucose update is unclear, conformational changes are known to trigger transport (66). The direction of glucose molecule migration during facilitated diffusion is governed by the relative concentration on the two sides of the lipid bilayer. In facilitated diffusion, the molecules are moved across the membrane with the aid of channel proteins and carrier proteins rather than dissolving in the lipid bilayer (67). When the concentration of glucose outside the cell declines, such as in liver cells where glucose is synthesized when blood sugar levels are low, the transportation of glucose via the lipid bilayer can also occur in the opposite direction. The largest superfamily of membrane transporters, which includes 74 families and more than 10,000 members, includes glucose transporters. The facilitative glucose transporters (GLUTs) and sodium glucose co-transporters (SGLTs) are two possible types of glucose transporters (68). The rate of pancreatic insulin production is inversely correlated with the rate of glucose diffusion. Glucose-6-phosphate is produced after diffusion into the cell. By doing this, a cell is equipped with an energy reserve that may be tapped into as needed by the body (69).

HepG2 cells were treated in media containing insulin in a study to determine the action and mechanism of diosgenin and 5-methoxypsorlen (5-MOP). The findings showed that glucose consumption decreased at high insulin concentrations (10^–6^°μmol/L) (70). However, the intake of glucose increased in a group of cells treated with diosgenin. The intracellular glycogen content also rose, showing that diosgenin was having a positive effect. During study related to action mechanism of diosgenin, it was revealed that the action of diosgenin significantly increased the phosphorylated expression of estrogen receptor-α (ERα), sarcoma (Src), Akt/protein kinase B, glycogen synthase kinase-3β (GSK3β), and the p85 regulatory subunit of phosphatidylinositol 3-kinase p85 (PI3Kp85x). In a group of cells that had received diosgenin treatment, the expression of GLUT-4 had significantly increased. Thus, it was possible to draw the following conclusions from the studies: diosgenin could reduce insulin resistance, increase glucose uptake, and hasten intracellular glycogen production. By encouraging the expression of adipocyte development in 3T3-L1 cells, diosgenin improves glucose uptake. The increased expression of mRNA also demonstrated its therapeutic potential (11, 49, 71). Diosgenin’s therapeutic potential was also examined in groups of 10 male Wister rats that had been given STZ. Sesame oil, diosgenin (10 mg), and diosgenin combined with a 5α-reductase (1 mg) inhibitor were administered to each diabetic group. Within 90−180 min, it was seen that the diosgenin-injected group’s blood glucose level had dropped. Diosgenin elevated serum dehydroepiandrosterone, which caused hyperglycemia in type 1 diabetic individuals. Diosgenin caused a 28% drop in glucose levels. The process is happening through the activation of AkT and PKC ζ/λ - GLUT4 signalling pathways. Both muscular AkT and PKC ζ/λ phosphorylation and GLUT4 translocation increased by 30% as compared to the untreated type 1 diabetic mice (64, 72).

Hepatic functions including gluconeogenesis and decomposition of glycogens also contribute to high blood sugar levels. These hepatic processes have been successfully controlled by diosgenin dose (73). The activity of glucose-6-phosphate dehydrogenase was dramatically decreased in the diet supplemented with 1% steroidal saponins from bitter yam or commercial diosgenin (74). Steroidal saponins possess a hexacyclic aglycone, such as diosgenin or tigogenin, in which the 3-OH group is decorated with an oligosaccharide chain. Also, diosgenin is a steroid saponin that is present in many plant species and is thought to provide a variety of essential therapeutic qualities. It is well recognized that glucose-6-phosphate plays a crucial function in controlling blood sugar levels while fasting. In glycolysis, glucose-6-phosphate is used to generate energy in place of ATP and NADH. Hyperglycemia has also been connected to a deficit in glucose-6-phosphate dehydrogenase (75).

## Insulin, insulin analogues, improved delivery, and insulin resistance

Insulin is a natural hormone playing a critical role in blood sugar level regulation and is formed by beta cells of Islets of Langerhans in the pancreas. Though the main function of insulin is the regulation of blood sugar levels, besides it is essential for certain metabolic regulations in the body including glucose storage control, protein and fat metabolism, and appetite regulation. In persons suffering from diabetes, either insulin is not produced by the body which is Type 1 diabetes, or the produced insulin is not used effectively by the body which is Type 2 diabetes (76). Insulin helps in the regulation of blood sugar levels by permitting sugar (glucose) from the blood to pass into the cells, where it is utilized as an energy source. During diabetes, the absence of insulin or its inability consequences in raised sugar levels in the bloodstream.

The artificial sorts of insulin being altered to enhance their pharmacokinetic activities are called Insulin analogs. They are produced to impersonate the normal insulin release from the pancreas resulting in improved glucose control in the blood in diabetic patients (77). Insulin analogs are produced to have advantages than traditional insulins including better absorption, extended action-time, and better flexibility. Insulin analogs are of a number of types including rapid-acting, short-acting, intermediate-acting, and long-acting analogs (78). Insulin which is rapid-acting works swiftly and should primarily be taken prior to meals in order to resistor sugar spikes post-meal blood. These insulins work within 15 min post administration and showed a crowning effect between 0.5−3°h and last for around 3−5 h. On the other hand, short-acting insulin also known as regular insulin (such as human insulin) works slowly comparatively and likewise is also given formerly meals. This starts working within 0.5°h after administration, peaks in 2−4 h, and lasts for 6−8 h. Insulin that is intermediate-acting shows an extended interval and is taken once or twice a day, having a listless action onset, typically 1−2 h after taking. Its peak effect is between 4−12 h which lasts for 12−18 h. Neutral Protamine Hagedorn is this type of insulin analogue (79). While long-lasting insulin delivers a sturdy insulin release for a prolonged time and is frequently administered as basal insulin. These insulins provide a basal level of insulin release over an extended period, usually covering 24 h or longer. They have a relatively stable effect with no pronounced peaks. However, considering an individual’s requirements, an amalgamation of diverse types of insulin might be given.

Maintaining sugar (glucose) levels in the blood, insulin therapy lessens the menace of severe snags and recovers long-term consequences in diabetic patients. By providing exogenic insulin, it recompenses for the insufficient formation or insulin utilization in the body. This assistance averts hyperglycemia and its related problems. Type 1 diabetes, a disorder identified as diabetic ketoacidosis may happen during severe insulin lack (80). Insulin therapy improves essential in diabetic ketoacidosis prevention. Giving sufficient insulin, energy, or fat breakdown can be prevented, which is usually responsible for ketones accumulation and blood acidification. Insulin enables glucose uptake by cells, letting it be cast off for the production of energy.

There are numerous approaches to the improvement of insulin delivery that aim to augment the absorption, administration, and glycemic control of insulin. Insulin pens are expedient devices using one-use insulin-filled cartridges and deliver insulin better than outdated vials and syringes. Insulin pumps deliver insulin continuously by using a catheter located underneath the skin, provide a stable basal insulin rate, and permit bolus dosages prior to meals (81). Jet injectors distribute insulin by using a high-pressure stream that infiltrates the skin, eradicating the use of needles. Inhalable insulin is a newfangled insulin delivery method using the device to deliver insulin in a powdered form and inhaled it into the lungs. Insulin patches stick to the skin and deliver insulin over microneedles or a permeable membrane and offer continuous insulin delivery (82). Although the abdomen is the furthermost site for the injection of insulin, alternate injection sites *viz.*, thighs, upper arms, and buttocks. Insulin resistance is a disorder where the cells of the body especially fat, muscle and liver turn out to be less receptive to the insulin effects (83). As an outcome, the pancreas produces more insulin is being produced to recompense for the diminished sensitivity which leads to advanced insulin levels in the bloodstream called hyperinsulinemia.

Diosgenin has been explored for its anti-diabetic activities and its latent to advance insulin sensitivity. Some reports have indicated that diosgenin enhances the uptake of glucose in the cells, improves insulin signaling, and ameliorates diabetes effects. It has been reported that administration of different diosgenin doses (15, 30, and 60 mg/kg body weight) daily to diabetic rats for 45 days caused a significant (*p* < 0.05) decrease in glucose levels in the bloodstream and an upsurge in plasma insulin. The transformed actions of key enzymes of carbohydrate metabolism in the muscle and kidneys were regressed significantly (*p* < 0.05) to almost normal levels (84) and the found outcomes were related to a standard oral hypoglycaemic drug. Diosgenin effect on the skeletal disarrays persuaded by experimental type 1 diabetes in 3-month-old female rats induced by single streptozotocin injection (60 mg/kg *i.p.*) was studied (85). Diosgenin (50 mg/kg/day) was given after 2 weeks till 4 weeks and found that diosgenin countered the diabetes effect on the growth and cancellate bone in the distal femur signifying positive impact on the skeleton. Recently, it has been studied that diosgenin attenuates non-alcoholic fatty liver disease in type 2 diabetes by regulating SIRT6-related fatty acid uptake in spontaneous diabetic db/db mice *in vitro* and *in vivo* (86). Fenugreek extract and diosgenin protected the liver against Non-alcoholic steatohepatitis, and diosgenin showed a dose-dependent impact. Though, the activation of the AMPK cascade was believed to be the mechanism for hepatoprotective effects (87). However, most of the investigation of diosgenin’s effect on insulin role is led in animal models or cell cultures. Partial clinical research is done in humans, and the consequences are not yet decisive. Additional examination is required to elucidate the exact actions or mechanisms and therapeutic potential.

## Promote adipocyte differentiation

Adipocyte differentiation is a process in which preadipocytes (precursor cells) progress into mature adipocytes having a tendency to store and release fat. In the milieu of diabetes, lessened differentiation of adipocytes may result in insulin resistance, a key indicator of type 2 diabetes, and led to raised sugar levels in the bloodstream and the pancreas generating more insulin in an effort to reimburse, ultimately causing dysfunction of pancreatic beta-cells. Numerous aspects including chronic inflammation, obesity, hormonal imbalance, and genetic malfunctioning can interrupt the normal adipocyte process differentiation leading to insulin resistance during diabetes (88). Visceral fat in the abdominal cavity is further active metabolically and related to a sophisticated risk of insulin resistance and diabetes related to subcutaneous fat under the skin. In diabetes, the study of molecular mechanisms and dysregulation of adipocyte differentiation is an active extent of research. By detecting vital aspects intricated in the process, investigators aim to advance new beneficial approaches for controlling diabetes and its linked difficulties. Despite the fact that diminished differentiation of adipocytes and insulin resistance are strictly associated with type 2 diabetes (89), type 1 diabetes is chiefly an autoimmune disorder when the immune system erroneously outbreaks and abolishes insulin-generating beta cells. Differentiation of adipocytes is not as much directly linked to type 1 diabetes, nonetheless, metabolic dysregulation and resistance of insulin may still happen in persons with this illness.

Diosgenin improves the metabolism of glucose by endorsing the differentiation of adipocytes and hindering inflammation in adipose tissues as reported in the literature. Diosgenin reduced the adipocytes and improved the expression levels of mRNA differentiation-related genes in adipose tissues, repressed penetration of macrophage into adipose tissues, and hindered expressions of numerous molecular components linked with inflammation in 3T3-L1 cells (90). Diosgenin weakened metabolic dysfunction in high-fat diet-fed mice, as demonstrated by declined glucose levels in the blood and improvement of glucose and insulin intolerance. Diosgenin repressed 3T3-L1 adipocyte differentiation, declined the size of adipocytes, inhibited PPARγ, and increased nuclear expression of Erβ which significantly suppressed diosgenin-exerted suppression of adipocyte differentiation and PPARγ expression indicating the repressive effect of diosgenin on adipocyte differentiation and validated that ERβ-exerted regulation of PPARγ expression and action is critical for diosgenin-inhibited adipocyte differentiation (91). 3T3-L1 adipocytes and RAW 264 macrophages evidently heightened tumor necrosis factor-α production, chemoattractant protein-1 monocyte, and nitric oxide, however, diosgenin treatment repressed the formation of these proinflammatory mediators and also blocked the inflammation macrophages persuaded from 3T3-L1 adipocytes. Also, diosgenin repressed the degradation of inhibitor κB and c-jun N-terminal kinase phosphorylation in macrophages and might be useful for amending the seditious changes in obese adipose tissues (92). Type 2 diabetes was induced in experimental animals by feeding high-fat diet (HFD) for 8 weeks followed by streptozotocin (STZ) injection (sub-diabetogenic dose; 35 mg/kg body weight). Oral administration of diosgenin at two doses (40 and 80 mg/kg body weight) for 14 days abridged hyperglycemia, hypercholesterolemia and hypertriglyceridemia and lipid accumulation in 3T3-L1 preadipocytes inveterate its adipogenic activity prejudiced by PPAR γ and PPAR α (93). Diosgenin lowers the damage of diabetes by altering cellular pathways for pancreatic β cell renewal for improved secretion of insulin and modifying ER-α-mediated PI3K/Akt pathways (94).

## Safety dosage of diosgenin

Diosgenin is the main component of fenugreek saponins and a secondary metabolite. At dosages of 1125 mg/kg and higher, steroidal saponins, which include diosgenin, exhibited deleterious effects and even death. Interestingly, the traditional steroidal saponins dosage is 510 mg/kg/day, implying that steroidal saponins, in combination with diosgenin, have no significant toxicity at this dosage. Male Wistar rats were given diosgenin in three different doses (1, 10, and 50 mg/bw kg, respectively) and fenugreek seed (0.2 g/bwkg) orally for 6 weeks. The rats given 1 mg/kg diosgenin and fenugreek seed had a higher insulin sensitivity index and a higher metabolic clearance rate (95). For 1°week, male F344 rats were fed 0 or 1% fenugreek seed powder (FSP) or 0.05% or 0.1% diosgenin before receiving azoxymethane (15 mg/kg body weight). Bioactive substances found in fenugreek seeds, including protodioscin, trigoneoside, diosgenin, and yamogenin, affect a number of enzymes, including those involved in glucose and lipid metabolism. Dietary FSP at 1% and diosgenin at 0.1% inhibited total aberrant crypt foci by up to 33 and 39%, respectively, during the promotional stage (96). The viability and growth of HCT-116 cells were reduced by diosgenin in a dose-dependent manner. After 24 h, the IC50 cytotoxic dose of diosgenin in HCT-116 was 35°M, while concentrations of 32°M or higher reduced the percentage viable cells by 50%. The viability and growth of HCT-116 cells were reduced by diosgenin in a dose-dependent manner. After 24 h, the IC50 cytotoxic dose of diosgenin in HCT-116 was ∼35°μM, while concentrations of ∼32°μM or higher reduced the percentage viable cells by 50%. Increasing diosgenin concentrations reduced HMG-CoA reductase expression at both the mRNA and protein levels. Food saponin, diosgenin, is a powerful inhibitor of HCT-116 human colon carcinoma cells, inhibiting growth and inducing apoptosis (97). In rats, a dose of diosgenin (40 mg/kg) protected against changes in liver markers and the antioxidant system of red blood cells without causing any side effects (98). Diabetic rats were given diosgenin (40 mg/kg bw) orally, which significantly reduced plasma glucose while increasing insulin levels. Diosgenin may play a protective role against aortic damage caused by oxidative stress in diabetics by modulating antioxidant defence and reducing lipid peroxidation in the aorta (99). These studies demonstrated that diosgenin and its derivatives are non-toxic, highlighting their utility in the treatment of chronic diseases.

## Conclusion and future prospective

Recent years have seen an increase in interest in using herbal medicine to supplement conventional therapies or treat a variety of conditions. The growing diabetic population, the demand for new medications from patients, and the potential market for new medications will all continue to drive fresh and creative research into all parts of diabetes therapy. The foundation for the subsequent generation of targeted drug development programmes has been laid by the mainstay medicines, which have been intensively researched throughout the years. Diosgenin, the primary active component of fenugreek, has been the subject of numerous tests by researchers looking at its role in the treatment of diabetes. Additionally, it is well recognized that the majority of diabetic problems are intimately tied to inflammation and oxidative stress in addition to impaired glucose and lipid metabolism. In addition to having an effective anti-inflammatory and antioxidant action, diosgenin also has a positive therapeutic effect on diabetes complications. Numerous studies have demonstrated the pharmacological advantages of diosgenin and its derivatives in the treatment of cancer, diabetes, osteoporosis, Alzheimer’s disease, and stroke. It has been demonstrated that diosgenin interacts with a number of molecular targets that are crucial actors in the occurrence and incidence of many major illnesses. Additionally, a multitarget medication strategy targeting various risk factors is a crucial paradigm and a cutting-edge technique for treating neurological diseases with complicated pathophysiology. To treat the various pathogenic characteristics of these disorders, combination therapies of diosgenin with substances exhibiting numerous modes of action are anticipated to be more effective than single medications. Therefore, it is strongly advised that future research use a systematic experimental design to evaluate the long-term effects of DG and/or its derivatives for the treatment of neurodegenerative illnesses and the management of associated symptoms. In-depth research must also be done on the risk assessment and safety evaluation of the pharmaceutical use of diosgenin or its derivatives in the treatment of neurodegenerative diseases.

## Author contributions

YT: Conceptualization, Writing – original draft. MK: Conceptualization, Writing – review & editing. AC: Writing – review & editing. MS: Supervision, Writing – review & editing. PV: Writing – review & editing. MB: Conceptualization, Writing – review & editing. CK: Writing – review & editing.
